# Cross-cultural patterns in mobile playtime: an analysis of 118 billion hours of human data

**DOI:** 10.1038/s41598-022-26730-w

**Published:** 2023-01-07

**Authors:** David Zendle, Catherine Flick, Darel Halgarth, Nick Ballou, Simon Demediuk, Anders Drachen

**Affiliations:** 1 Department of Computer Science, University of York, York, North Yorkshire UK; 2 Centre for Computing and Social Responsibility, De Montfort University, Leicester, Leicestershire UK; 3 School of Electronic Engineering and Computer Science, Queen Mary University of London, London, UK; 4 Digital Creativity Labs, University of York, York, North Yorkshire UK; 5 Faculty of Engineering, University of Southern Denmark, Odense, Denmark

**Keywords:** Human behaviour, Health policy

## Abstract

Despite the prevalence of gaming as a human activity, the literature on playtime is uninformed by large-scale, high-quality data. This has led to an evidence-base in which the existence of specific cultural gaming cultures (e.g. exceptional levels of gaming in East Asian nations) are not well-supported by evidence. Here we address this evidence gap by conducting the world’s first large-scale investigation of cross-cultural differences in mobile gaming via telemetry analysis. Our data cover 118 billion hours of playtime occurring in 214 countries and regions between October 2020 and October 2021. A cluster analysis establishes a data-driven set of cross-cultural groupings that describe differences in how the world plays mobile games. Despite contemporary arguments regarding Asian exceptionalism in terms of playtime, analysis shows that many East Asian countries (e.g., China) were not highly differentiated from most high-GDP Northern European nations across several measures of play. Instead, a range of previously unstudied and highly differentiated cross-cultural clusters emerged from the data and are presented here, showcasing the diversity of global gaming.

## Introduction

Mobile games comprise the single largest segment of the global games market. Estimates have indicated that over 2.8bn people play mobile games^1^, downloading more than 80 billion mobile games annually^2^, and that mobile gaming accounts for at least half of total games-related revenue^3,4^. Despite the prevalence of mobile gaming, little is known about how mobile playtime varies around the world. The prior evidence base regarding cross-cultural differences in gaming has been limited by three key issues: (1) a lack of large-scale cross-cultural data; (2) a lack of representative samples; and (3) a lack of behavioural data.

Firstly, previous academic attempts to map cross-cultural differences in gaming have tended to focus on small groupings of nations. For example, in^5^ researchers investigated differences in gameplay between the United States and Poland across a variety of platforms, including mobile, PC, and console. They found several such differences—for example, that playing multiplayer games with participants physically co-present may be more popular in the United States than in Poland. However, the scope of their data limited their inferences to these two specific nations. Similarly, in^6^, researchers investigated the relationship between gaming on smartphones and problematic smartphone use. They found few cross-cultural differences in these factors between Belgian and Finnish gamers; however, the scope of this work was limited to Belgium and Finland, and thus results could not generalise globally. In the same vein^7^, investigated how social gameplay changed across cultures during the pandemic: However, participants were drawn from small European and Indian samples, and hence could not generalise beyond these nations. Similarly, in^8^, researchers investigated factors underpinning the adoption of mobile gaming in South Africa and Norway. Their results suggested that factors underpinning such adoption (e.g. service cost) differed between South Africa and Norway; however, their results were unable to generalise more broadly than this. Such a trend may also be observed within literature dealing with cross-cultural adoption of technology more broadly. For example, in^9^, researchers investigated the intention of individuals to engage with commerce using a mobile device via self-report survey: However, to do so, they used a sample drawn from two specific countries.

Secondly, prior work that deals with the nature of gaming within or between cultures tends to lack access to representative samples, and cross-cultural differences that are inferred from this work may therefore prove unreliable. For example, in^10^, researchers investigated differences in gameplay between Japanese and British adolescents. They found that Japanese participants tended to game for longer than those in the UK—however, the ability of their sample (204 12–14 year olds from London and 305 12–13 year olds from Tokyo) to generalise to wider populations of Japanese and British adolescents is uncertain. Similarly, in^11^, researchers investigated whether individuals differed between countries in how they played *Battlefield 3*. They found numerous such differences—for example, that German and Swedish players demonstrated cooperative behaviour in-game more frequently than US or UK gamers. However, this study used convenience sampling, and the representativeness of the within-country group data (and thus the generalisability of any between group comparisons) is unclear. Such issues are commonplace in the literature on cross-cultural technology use: In^12^, for example, researchers investigated how patterns of mobile phone usage might vary between Sweden, the USA, and Japan—and explicitly drew attention to the difficulty in finding representative or comparable samples across these groups.

Thirdly, a lack of data sharing from industry sources has meant that prior research into the correlates and distribution of playtime has largely relied on self-report or unclear measures of this factor. For example, in both^13,14^, researchers investigated the demographic predictors of high gameplay in samples of German adults and adolescents. In both cases, playtime was measured via self-report; in both cases, a gender effect emerged in which males tended to play for longer than females. However, recent work has problematised playtime research which bases its measurement strategy in self-report. Such research has highlighted the existence of discrepancies between how much time individuals report spending using technology, and how much time a device objectively logs them as spending^15^. Indeed, this weakness of correlation between logged and reported behaviour has been observed in the playtime domain in specific^16^. Similarly, substantial grey literature exists which attempts to predict or describe the uptake of gaming—and mobile gaming in specific—across the globe. However, the data generation process that lies behind such reporting is often unclear, and thus too is the confidence that researchers may place in it. For example^17^, consists of a market overview produced by the professional services brand PricewaterhouseCoopers. It reports and forecasts playtime and revenue across a variety of territories and products: However, the methodological basis for these estimates is unavailable for scrutiny. Similarly, in^18^ is a business intelligence report that summarises growth in mobile gaming across Africa; however, its methodology is unclear and its accuracy is hence also unclear.

Research which is based on the usage of secondary video game telemetry data may address the issues outlined above. Broadly referred to as game analytics, a variety of studies utilise remotely-collected data from video games, along with associated quantitative data sources, to understand player behaviour in games^19,20^. The focus of this work varies depending on the domain that the telemetry data is being applied to inform, and includes AI development, prediction modeling, behavioral profiling, studies of health and gameplay, as well as research into the effects of gaming on society and wellbeing^16,20–25^. However, as with the evidence base outlined above, previous game analytics studies are limited in their ability to represent both the world and the diversity of modern games: previous analyses of playtime’s significance in society have tended to use playtime data from one or a few games, and usually in restricted samples^22,23^. In game analytics, the largest telemetry-based studies available cover a few hundred to a few thousand games, and use data from a single platform—typically *Steam* (www.steampowered.com)—which provides some basic information about the games on its platform and minimal geographical information^23,25^.

This lack of insight into cross-cultural differences in playtime has important ramifications for decision-making in both political and industrial spaces, as well as in video game research^26^. Given the size of the global games industry and the diversity of its customer base, and the ongoing debates around games and their possible influences—beneficial or harmful—on those who play them, this leads to an important knowledge vacuum^16,25,27,28^. In the absence of data and analysis which overcomes the challenges detailed above, decisions risk being guided by claims of unclear substantiation. For example, beliefs about excessive gaming being particularly prevalent amongst East Asian populations has informed decisions to implement laws limiting game use for children in China^29^ and previously South Korea^30^. Such policymaking should ideally be informed by the highest quality possible evidence regarding playtime. Similarly, industrial decisions about where to release games may be influenced by perceptions of habits in different regions: given that localisation can be a costly investment, games may not be released in areas perceived to play games less, or be less interested in particular game genres or mechanics. Therefore, there is a need for research illuminating gaming habits across the globe, for mobile games as well as other game formats.

### The present research

*Unity Technologies*, which supports the most used game engine worldwide, has provided access to de-identified and anonymised playtime and geographical data from every mobile game for the period 1st October 2020 to 1st October 2021. The dataset used covers about 5 billion days of playtime (roughly 329 million hours of play per day in the year).

While it is unknown exactly how large a fraction of the mobile games market relies on the Unity game engine, Unity Technologies’ own estimates (from 2019) indicate that 53% of the top 1000 mobile games on the iOS App Store and Google Play were made with the Unity engine^31^. Industry-based reports also highlight Unity’s dominance, e.g., for the UK^32^. No claims are here made outside the data analysed, however, even within the confines of the dataset, the goal is to illuminate global cross-cultural variation in playtime and play behaviour on a much larger scale than previously possible. As the dataset only includes mobile titles, no conclusions are here drawn for the PC or console market, and the cross-cultural patterns for these games may be different than what is presented here.

In this paper, we describe a global dataset of mobile gameplay provided by *Unity Technologies*, and the national and cross-cultural patterns in the market. Towards exploring cross-cultural patterns further, we perform a cluster analysis across 214 separate countries and regions, focusing on three key features and thus providing a multivariate view on how mobile games are played across the world: yearly hours of playtime per capita (HPC); minutes of average daily playtime per engaged user (MDP); and playtime inequality—the proportion of playtime that comes from the top 1% of players (PI). This establishes a data-driven set of groupings and define similarities and differences in how the world plays mobile games.

The work presented substantially advances the state-of-the-art of knowledge about global patterns of mobile gameplay, notably in terms of moving from smaller-scale studies in terms of number of games, to millions of game titles and a global area scale. The results presented identify a number of highly differentiated global gaming cultures which have not been previous identified or studied (e.g., island-based nations with high playing activity).

## Method

### Research questions

Overall, this research aims to explore whether broad cross-cultural groupings in play exist. Thus, we define RQ1 as:

RQ1: “What natural groupings exist regarding the way that different countries play games?”

An additional aim of the research is to specifically investigate whether East Asian countries tend to form a monolithic bloc in terms of how they play games, as suggested by techno-Orientalist accounts of gaming. Thus, we define RQ2 as:

RQ2: “Do East Asian countries tend to be highly differentiated from the rest of the world in terms of playtime?”

### Data and preprocessing

Unity Technologies provides solutions for game developers to collect behavioural telemetry from games based on the Unity Engine. The flow of data basically originates at the client—the mobile platform of the user. Telemetry data from video games is, similar to other user-facing applications, collected from the installed clients, and transmitted to cloud solutions depending on the size of the data involved. Here the data are masked and filtered for compliance, before being analysed for ethics and data collection. From storage, data is extracted and analysed either in the cloud or locally, and in this case made available to the research team^20^.

Unity Technologies thus capture data directly from user devices, as is the current norm for mobile applications. While basic platform, location and other technical information is recorded from all games made with the Unity Engine (for details, see: https://unity3d.com/de/legal/privacy-policy), the *Unity Analytics* toolkit provides an additional broad range of opportunities for developers to integrate behavioural telemetry tracking in their games, for example recording transactional data, which is a standard practice across the Creative Industries^22^. While it is in theory possible for a Unity game to not have Unity Analytics enabled, this is rarely the case for the simple reason that a substantial number of basic game functionality cannot operate without data collection about user behaviour. For example, it is not possible to measure player progress, generate save points, handle equipment management, support purchases, etc., without capturing some player data.

If a user does not have an internet connection, a buffer system stores data locally on the device, until connection is re-established. Furthermore, for Unity Analytics, if a user should leave an active game running but not interact with it, a timer (20 min) sets in which stops collecting data for the active session. Games running in the background are not integrated in session time.

The data used for this study was telemetry data from mobile games only. Games for other platforms—the Unity game engine supports over 20—as well as productivity applications and other types of software were removed. The subset of the data used in this paper dates 1st October 2020 to 1st October 2021, to provide the most recent year possible worth of data. The dataset contains information from (2,189,789) distinct titles. Access was provided to this data within a Google Cloud Platform *BigQuery* (an SQL variant) table, containing one row for each user’s daily usage of a specific game. Raw data contained daily information about each individual who had played a mobile game, made using the Unity Engine with Unity Analytics enabled. This data consisted of: (1) an anonymous but unique identifier for a user; (2) an identifier for the country/region in which a user’s play took place; (3) an identifier for the game that was played; and (4) the total duration of playtime on that day for each game. Users were uniquely identified within games, but not across games. It is therefore possible for players to engage with multiple mobile games at the same time and be counted multiple times. More than 52 billion unique identifiers are present in the data.

The dataset contains data on a total of 118 billion hours of recorded playtime. Average duration of play per country is 553 million hours but with an SD (Standard Deviation) = 1907 million hours indicating wide differences across countries for the timeframe. Each session is linked to a country or region using the user’s IP. Locations are mapped to places with an ISO-3 code, which includes certain territories and disputed regions such as Guam; for this reason, we use the term “countries and regions” or “territories” here (sometimes shortened to “regions”). This link procedure is carried out by Unity Analytics, and the research team did not have access to any personally identifiable information (see “Ethics of data collection” section below). There are 250 countries and regions represented in the Unity data. 214 of these were retained following initial data pre-processing. The World Bank dataset that we used provided population data on 215 of these areas. The data from Cuba proved anomalously scarce with many missing entries, and was therefore removed from our dataset prior to analysis, leaving us with 214 total countries or regions.

For the analyses presented here, the data was aggregated and processed at the region level using *BigQuery* before being exported to R and Python for analysis and visualisation. The duration of all sessions that occurred in a given country or region were summed, and the number of sessions recorded.

### Negotiation of data access

The data used for this study is not publicly available due to their commercial sensitivity. Access to an anonymised and pre-processed subset of Unity’s data lakes was given to the first and last authors of this paper by a technical team at Unity. Whilst Unity’s overall datasets remain private, all data that were used to generate the models contained within this paper have been made public and can be found at the OSF link associated with this study.

### Ethics of data collection

Unity Technologies collects a substantial amount of data from players using the Unity Analytics toolkit, which is an extension that needs to be enabled by developers using the Unity Engine in order to operate. All Unity games, or games using Unity components, that integrate Unity Analytics prompt a consent agreement when installing the game that explains the collection and use of this data to the player. Unity Technologies has a set of documentation that explains the requirements for collection, storage, and use of analytics data to the developer (Unity Software Inc. 2021, see also: https://unity3d.com/de/legal/privacy-policy). One of the uses explained in the collection agreement is for research purposes, which is the purpose under which this data has been shared with the researchers. The specific data analysed here is not personally identifiable information (PII) and the research team does not have access to PII from Unity Analytics. The data presented here is aggregated from a collection that is pseudonymised by way of a token unique to each player of each individual game—no players are traceable across games, only within any specific game. Therefore, we consider this use of data within reasonable ethical use under research ethics norms and expectations. The research has also received IRB approval from the University of York Physical Sciences Ethical Review Committee.

All methods were carried out in accordance with relevant guidelines and regulations. All relevant analytic protocols were approved by the ethics committee outlined above at the lead author’s host institution (University of York).

This is not an experimental study and does not involve participants. It purely involves the secondary analysis of large, anonymised, pre-existing datasets. As such, no informed consent procedures were relevant to the work conducted here.

### Analytic approach

In order to explore and define any patterns in the playtime data across countries and territories, we utilized unsupervised machine learning, specifically cluster analysis^23,33^. The goal of this analysis was to identify countries, whose populations exhibit similar patterns of play behaviour in terms of how they play the mobile games in the dataset. This is not an analysis of which specific games were popular in different parts of the world, but rather an analysis of differences in the volume, intensity, and concentration of playtime itself in n the areas under analysis.

#### Measures

To describe playtime behaviour, three aggregate measures were defined: (1) hours of yearly playtime per capita (HPC); (2) minutes of average daily playtime per active user (MDP); and (3) playtime inequality (PI). These features are selected to describe three fundamental aspects of play: The first describes the *volume* of overall play within a country. It forms the starting point for investigating playtime in an area. However, volume of play does not characterize how much time the average player tends to spend in-game. Therefore, minutes of average daily playtime per active user is included as a measure of the *intensity* of play. The third measure—the playtime inequality (PI) index—describes the degree to which playtime is *concentrated* in a population. Does every gamer in this country play mobile games an approximately equal amount? Or is most playtime accounted for by a small but extreme subset of gamers? Jointly, these three measures form a starting point for analysing playtime-based behaviour. Each measure is defined as follows:

1. *Hours of yearly playtime per capita (HPC)* (Mean: 20.20 h, SD: 16.43 h): This feature estimates how commonplace mobile gaming is within a country in terms of volume of play. We divided the sum duration of playtime within a country/region by its population as estimated by the World Bank (most recent figure available).
2. *Minutes of average daily playtime per active user of the country/region (MDP)* (Mean: 13.93 m SD: 4.43 m): In order to estimate how intense mobile gaming was within a country or territory, we calculated the average amount of time that a user spent playing a mobile game each day: lower volumes of average daily engagement would indicate more distributed playtime behaviour. For each player in the dataset, we measured when they first were seen playing a game; when they were last seeing a game; and then divided their total playtime by this span of days.
3. *Playtime inequality of the country/region (PI)* (Mean: 44.83%, SD: 8.41%): In order to estimate how equally play was distributed within a country/region, we calculated the percentage of a region’s total playtime which came from the 1% of the players in terms of playtime (i.e., users with the top 1% most playtime of all users from that region) for the given time segment. This statistic allows us to evaluate the extent to which playtime is concentrated in a heavily engaged component of the population.

#### Clustering approach

In the context of customer behavior analysis in the games industry (and wider Creative Industries), machine learning can be used for a variety of purposes, including to reduce the dimensionality of a dataset in order to find the most important features, and locate patterns which are expressed in terms of user behavior as a function of these features, which can be acted upon, whether for the purpose of understanding markets, customer behavior, testing/refining game design or any of a host of other purposes including prediction^19,20,22,34^. Specifically, unsupervised machine focuses on fitting a model to observed data. However, unlike supervised machine learning, there is no a priori output. Input data are treated as random variables, and a density model built for the data. For example, in categorising countries according to the gaming behaviour of their populations, and we do not know these categories in advance (because we are observing a new phenomenon) we can use unsupervised learning techniques to define or learn the categories from the data^33^.

Unsupervised clustering techniques vary, from clustering algorithms such as k-means and c-means, to lower rank methods such as principal components analysis (PCA)^35,36^ and Non-negative Matrix Factorization (NMF)^37,38^, as well as archetype analysis (AA)^34,39^. Methods for unsupervised learning include self-organizing maps (SOM), which were used in the earliest telemetry-driven player classification studies in games^40^. Using approaches such as v-fold cross validation that adds a quasi-supervised component to cluster analysis, and can be useful in specific circumstances, especially when data are sparse or limited, which is not the case here^41,42^. Furthermore, interpretation of clusters can be difficult, especially in situations where cluster separation is not straightforward^33,43^.

A key assumption in clustering^33^ is that the behavior telemetry data can be stored in a *d* × *n* matrix ***V*** = *[v*_*1*_*,…,v*_*n*_*] ∈ R*^*d*×*n*^ so that each column corresponds to a specific entity (in this case, country/territory). Fundamentally, when considering a situation where *n* samples of *d-*dimensional vectorial data are gathered in a matrix ***V***^*d*×*k*^, the problem of determining functional clusters corresponds to finding a set of *k* << *n* centroid vectors ***W***^*d*×*k*^. Given that membership of the data points ***V*** to the centroids in ***W*** is expressed via a coeffient matrix ***H***^*k*×*n*^, clustering can be defined as a matrix factorization problem with the aim is to minimize the expected Frobenius norm ║***V − WH***║. Methods such as PCA, NMF and k-means try to minimize the same criterion, however, they impose different constraints and therefore yield different matrix factors^36,44^. As an example, NMF assumes ***V, W***, and ***H*** to be non-negative matrices and often leads to sparse data representations. PCA constrains ***W*** to be composed of orthonormal vectors and produces a dense ***H***, where k-means clustering constrains ***H*** to unary vectors. K-means is perhaps the most widely adopted unsupervised clustering algorithm across domains, as well as in game telemetry analysis where it is used to find central tendencies in behavioral data^20,34,45^, however, it is focused on retrieving compact cluster regions, and can in some cases be difficult to interpret^33^. All cluster models have different sets of advantages and drawbacks, which means a common strategy involve training a number of models with different search optimizations, and evaluate the fit of these individually, as is the case here (see below)^23,43^.

**Figure 1:**
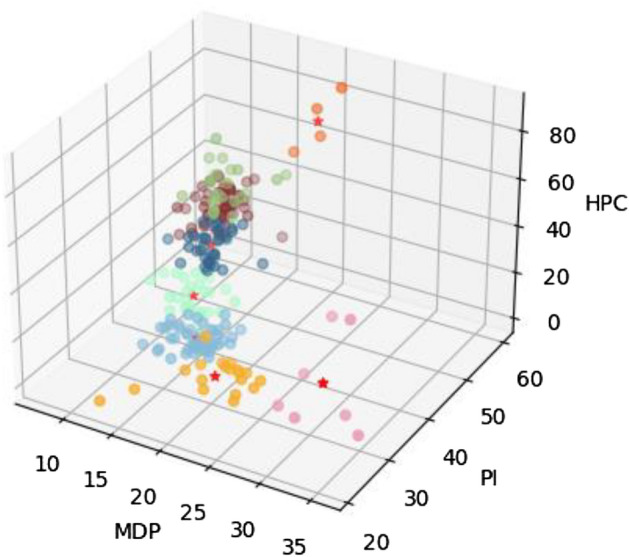
Unscaled (non-normalised) 3D scatterplot of the *k*-means cluster solution showing eight clusters. Red stars are cluster centroids (due to the 3D nature of the plot, not all cluster centroids can be seen in the plot). Colours correspond to Fig. 6.

We applied 4 different clustering algorithms on normalised data (data were normalised to a 0–1 scale) the data: K-means^46^, DBSCAN^47^, OPTICS^48^, and HDBSCAN^49^ in order to ascertain the best model for the dataset. We found that only the K-means approach was able to find a cluster solution, whereas all the other methods could not find a cluster solution without classifying some of the data points as noise (these models are particularly useful for finding densely structured clusters). As we know that there is no native noise in this dataset, we proceeded with the K-means approach. K-means is a method of vector quantization^46^. The model partitions the observed data points in multivariate space into a number of clusters (k). The algorithm partitions the data so that all points in a given cluster are closest to the corresponding cluster centre, or centroid, which may not overlap with an observation. The K-means model seeks to minimise squared Euclidean distances within clusters. As a cluster model, *k*-means focuses on central tendencies, as opposed to models such as Archetype Analytics, which seeks to uncover extremals in the data space^34,39^. The cluster solution is presented in Fig. 1, with centroid details in Table 1.

**Table 1:** Description of cluster centroids.

Cluster	MDP	PI (%)	HPC	Number of countries
A-Type	10.36	54.33	29.03	51
B-Type	15.18	37.33	3.61	47
C-Type	12.66	46.68	26.31	36
D-Type	12.8	42.04	11.75	27
E-Type	12.0	51.58	44.05	23
F-Type	19.65	31.73	2.03	19
G-Type	30.03	33.13	10.38	7
H-Type	19.20	57.70	70.66	4

MDP = minutes of average daily playtime per user; PI = playtime inequality, i.e., the percentage of a region’s total playtime which came from the top 1% of players; HPC = hours of yearly playtime per capita. Cluster labels after representative animals from the specific regions. Cluster labels do not imply a ranking or commentary but is purely descriptive with animal size a rough indicator of total play per capita for the cluster.

Determining the best number of clusters for a given dataset is a process that inherently carries with it an element of human decision-making, as do all unsupervised learning methods^33,43^. While in practice it is possible to rely completely on techniques such as elbow plots or silhouette plots determine the number of clusters, applied clustering usually involves a trade-off between the mathematically best fit and the explainability and usefulness of the underlying clusters^33,43,50,51^. In the context of video games, this is described in detail by^34^. In the current case, it was chosen to rely fully on modelling, to avoid any implied bias. The cluster solution is presented in Fig. 1.

In order to determine the correct number of clusters to use with the *k*-means algorithm we applied the Kneedle algorithm to the elbow plot^52^: The elbow plot is a plot of the distortions of the clusters, in this case we used the average Euclidean distance between the data and their corresponding cluster centroids for various *k* values. It is common practice when using the *k*-means algorithm to pick a *k* value at which the distortions curve flattens out; this is called the elbow. The Kneedle algorithm can be used to algorithmically identify the “knee” point in a curve or in our case the “elbow”^52^.

## Results

**Figure 2:**
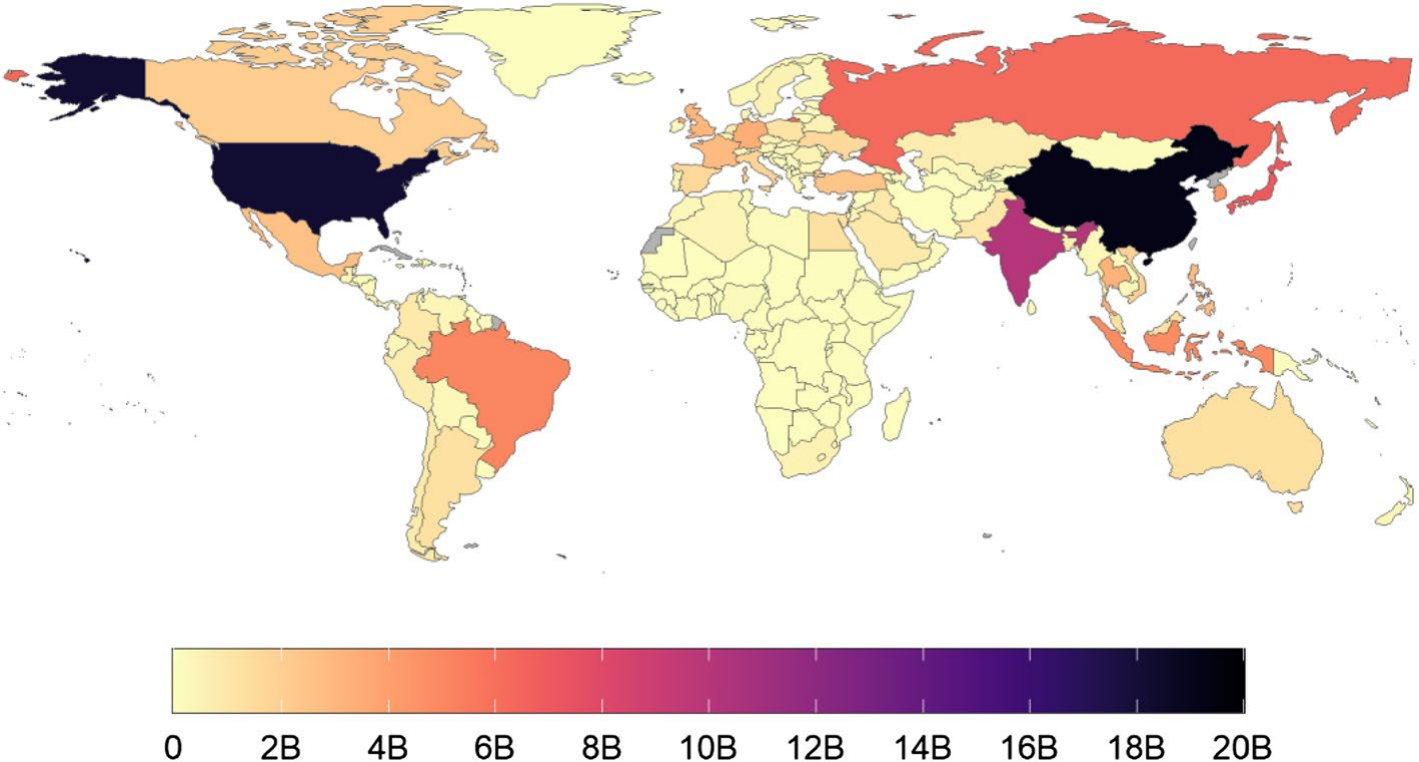
Heatmap of total mobile gaming hours, Oct 2020–2021. B = Billion. Countries/regions with no data in grey colour. This map was generated using ggplot in R (v4.0.2). Code for reproducing these maps is available at the OSF repository associated with the project.

**Figure 3:**
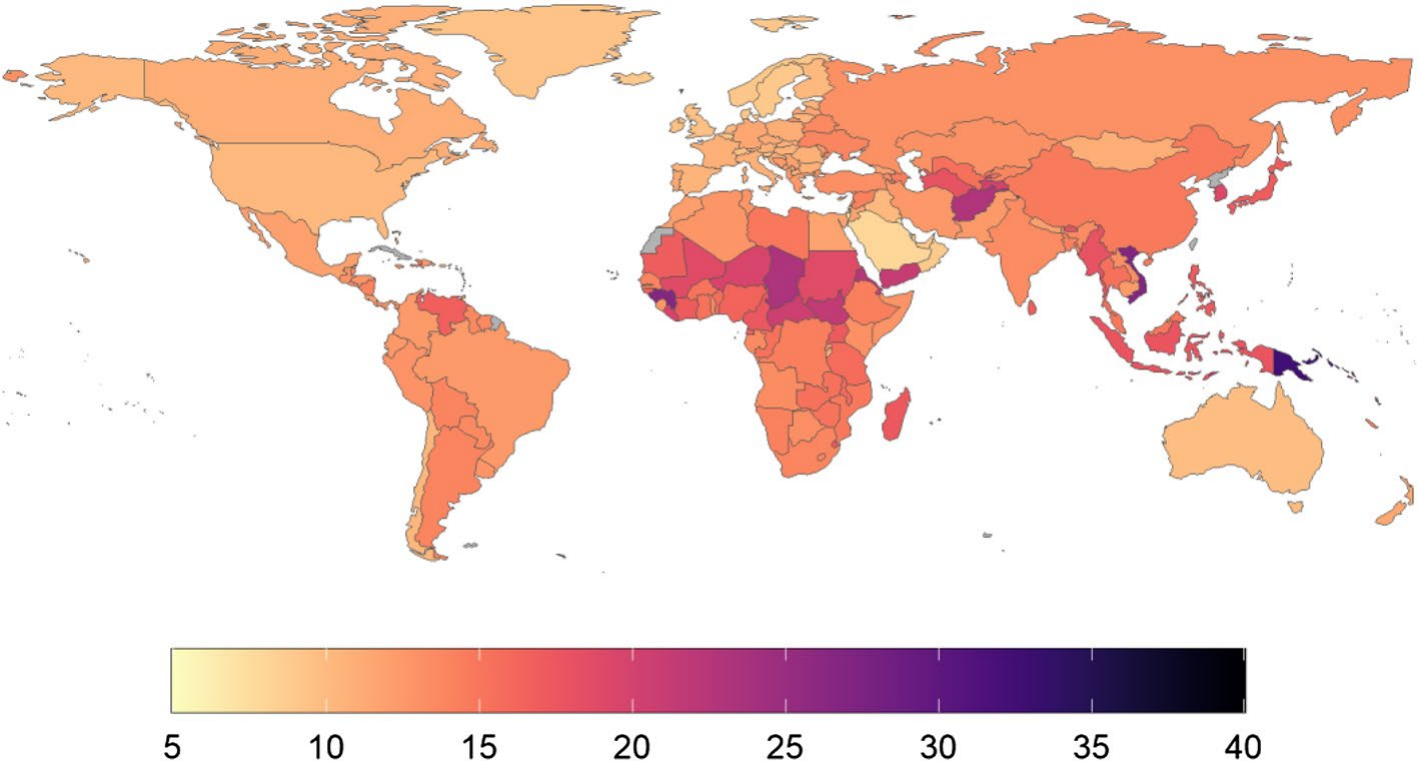
Heatmap of minutes of average daily playtime per active user (MDP), Oct 2020–2021. Countries/regions with no data in grey colour. This map was generated using ggplot in R (v4.0.2). Code for reproducing these maps is available at the OSF repository associated with the project.

**Figure 4:**
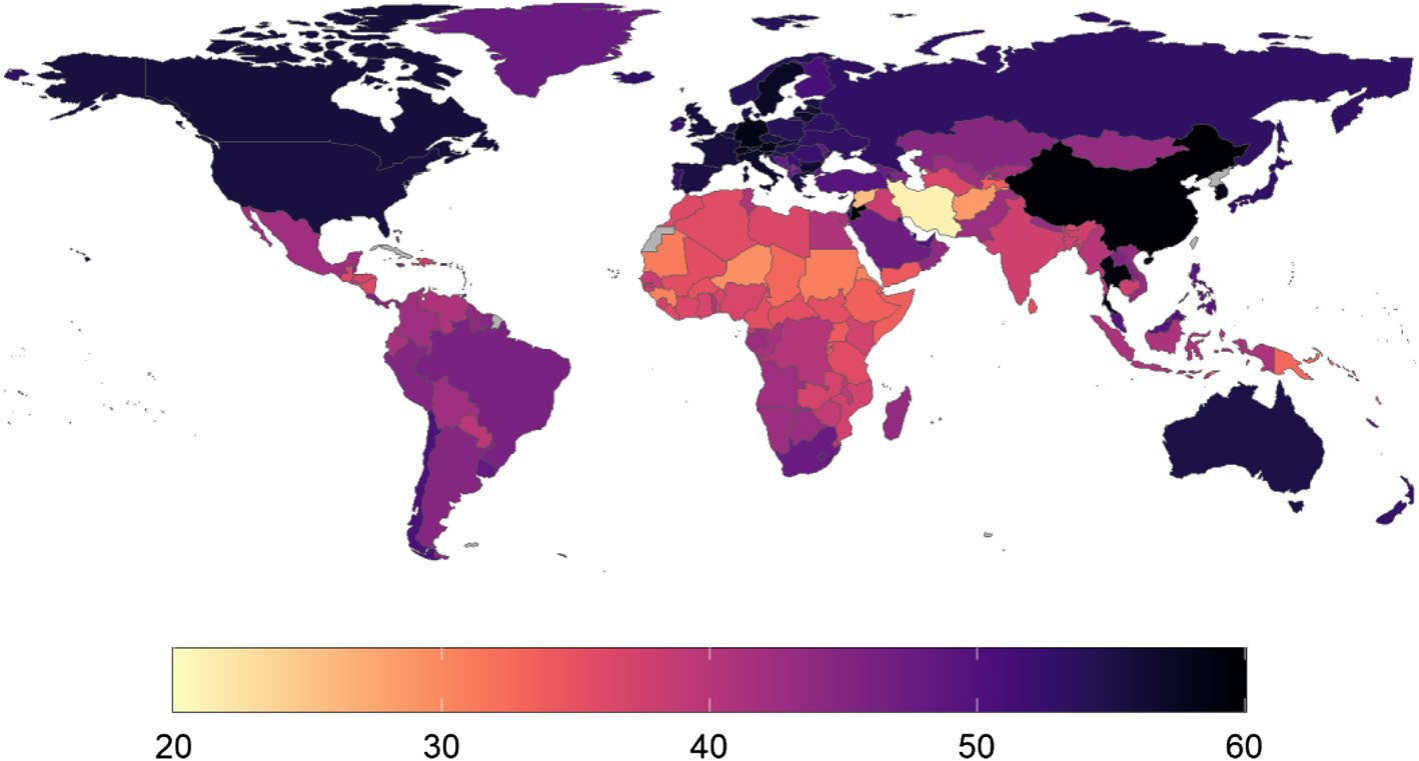
Heatmap of play inequality (PI), Oct 2020–2021. Countries/regions with no data in grey colour. This map was generated using ggplot in R (v4.0.2). Code for reproducing these maps is available at the OSF repository associated with the project.

**Figure 5:**
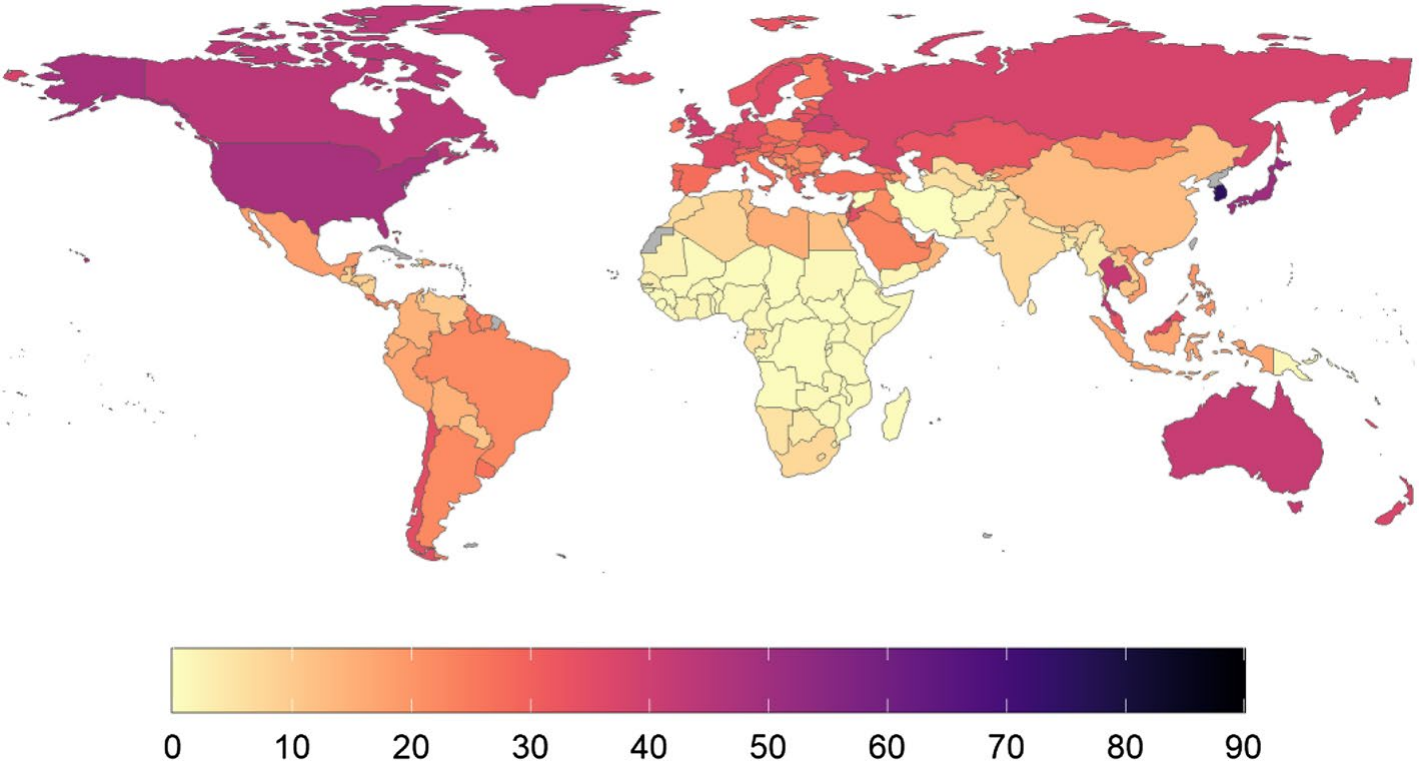
Heatmap of hours of yearly playtime per capita (HPC), Oct 2020–2021. Countries/regions with no data in grey colour. This map was generated using ggplot in R (v4.0.2). Code for reproducing these maps is available at the OSF repository associated with the project.

Data were clustered along three axes: Hours of yearly playtime per capita (HPC); minutes of average daily playtime per active user (MDP); and playtime inequality (PI). We present a map of total global playtime as Fig. 2. Maps describing the global distribution of each of the factors described above are presented below as Figs. 3, 4 and 5.

### Descriptive statistics

In terms of total playtime, China (19.2 billion hours), the United States (18.2 billion hours), and India (10.1 billion hours) logged the greatest amount of time playing Unity Analytics-enabled mobile games, with those three countries accounting for 35.4% of the total playtime across the world (Fig. 2). In terms of playtime per capita, Asia is home to five of the top seven regions in the world (Fig. 5): namely Hong Kong (89 h per capita), South Korea (75 h per capita), Singapore (62 h per capita), Macao (55 h per capita), and Japan (52 h per capita). Hitherto undescribed in the literature, the other two highest-playing regions were Guam (75 h per capita), and Barbados (56 h per capita).

By contrast, 34 regions had less than one hour per capita played across the entire 12-month period. This includes 30 regions in Africa as well as Syria and Iran. It is possible that for some of these countries, data may be missing due to limited internet infrastructure, internet shutdowns, restricted game availability, or other geopolitical factors. Additional work would be needed to explore these factors.

The most casual players in the world appear to come from high-GDP Middle Eastern nations. Four of the five lowest-scoring countries in the world in terms of average minutes of daily playtime per user (Fig. 3) are drawn from this geographical region: Kuwait (7 min), Israel (7 min), Saudi Arabia (7 min), and Bahrain (7 min). Contrastingly, countries where an average mobile player tends to spend longer in-game every day tend to be island nations. The top four countries in the world in terms of average minutes of playtime per player (active user) are Kiribati (35 min), Papua New Guinea (32 min), Nauru (32 min), and Tuvalu (30 min).

High levels of playtime inequality (Fig. 4) feature in both East Asian countries and small, above-average-GDP European countries: The majority of playtime in China (59.7%), Thailand (58%), Macao (57%), and Korea (58%) comes from the top 1%, but this is also the case in Germany (58%), Austria (57%), Sweden (57%), and Liechtenstein (57%). Countries where playtime was relatively egalitarian tended to be low-GDP countries with relatively low levels of overall playtime per capita. This group included several countries that are undergoing conflict: for example, Syria (25%), and Afghanistan (28%). These all feature some of the lowest levels of playtime inequality on the planet.

**Figure 6:**
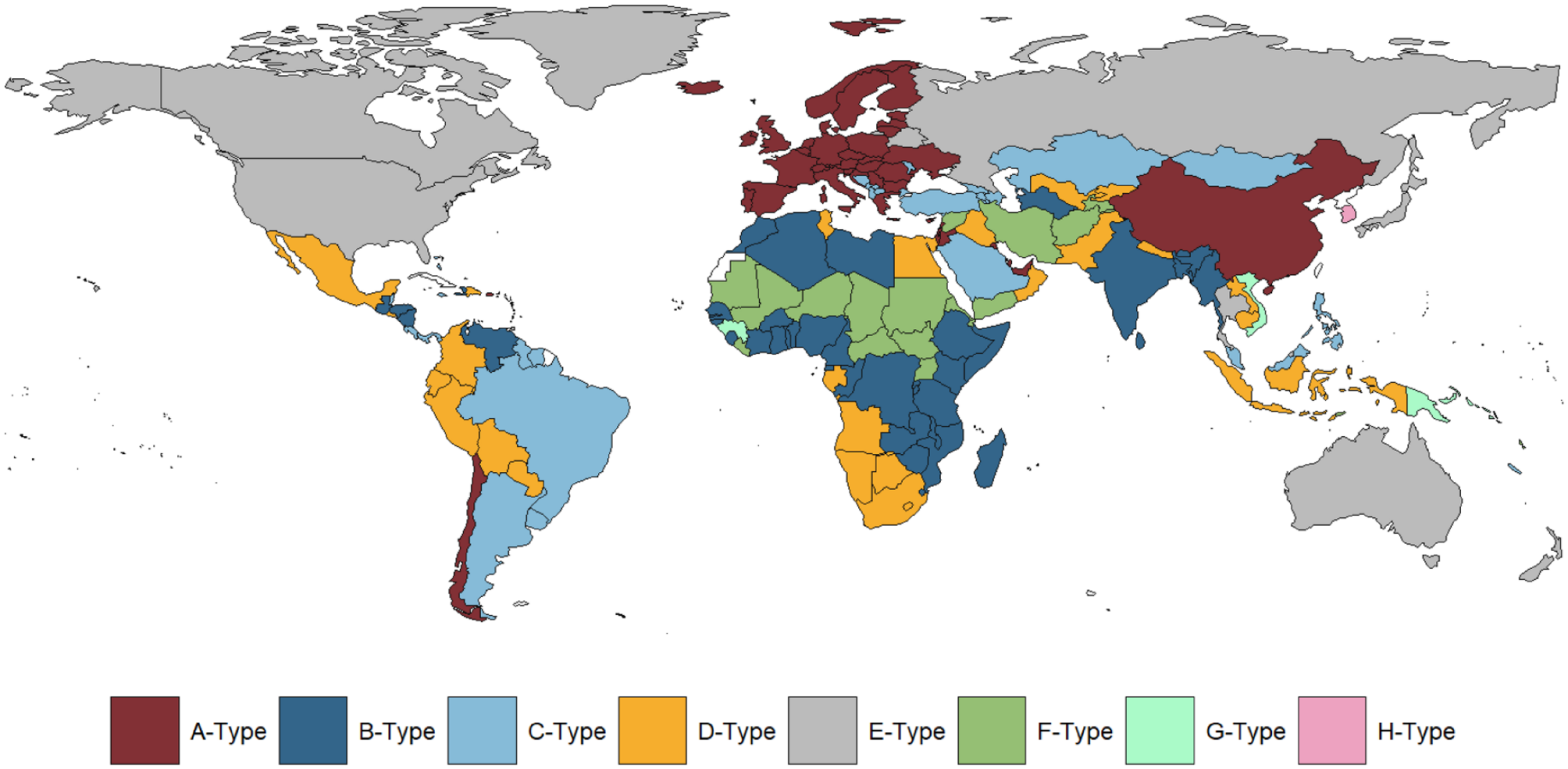
Global cluster membership on the basis of a combination of HPC (hours of playtime per capita), MDP (minutes of daily playtime per active user), and PI (playtime inequality). Map was generated using ggplot in R (v4.0.2). Code for reproducing these maps is available at the OSF repository associated with the project.

Frequency plots showing the distribution of both HPC, MDP, and PI are presented below as Figs. 3, 4 and 5. Core statistics for these metrics are reported below as Table 1. All show substantial variance across the 214 countries in the sample. 43 countries had a HPC of less than one hour (Fig. 6). For HPC and MDP, a handful of countries comprise a marked upper range outlier, corresponding with Cluster 4 (“Cluster analysis: play behaviour” section). MDP has the majority of the countries in the sample seeing daily playtimes of 9–17 min, suggesting this range encompasses most mobile gamers. PI is more evenly distributed across the range than HPC and MDP, topping out at almost 60% for China.

### Cluster analysis: play behaviour

The clustering approach outlined above resulted in the separation of 8 distinct clusters. The outcomes of this cluster process can be seen in Fig. 6, with descriptions of the cluster centroids in Table 1. Due to the complex nature and composition of the clusters, these are not given behaviourally descriptive labels, as these could be too cumbersome for writing. Instead, clusters are labelled from A-H in descending order depending on cluster size (I.e. A-Type countries fall within the largest cluster; H-Type countries fall within the smallest cluster).

*The A-Type Cluster* (51 countries/territories) is characterised by some of the world's most unequal gaming cultures: A-Type countries have a very strong layer of extremely engaged players overlaying their normative moderate mobile scenes (mean PI = 54.33%, China ranging above 59% followed by Germany at 58%). A-Type, as a cluster, is largely made up of European countries, along with small high-GDP areas within the Middle East and Asia. China is included in this cluster, and it would seem from the analysis that the nature of play in China is more similar to that of Europe and parts of the Middle East than it is to other high GDP Asian nations known for their video game culture like South Korea^53^. Outside of the high concentration of playtime in a small group of heavy players described above, mobile gaming tends to only be moderately common in A-Type countries: these regions display mid-range duration per capita (mean = 29.03 h), and mid-range average play per day (mean = 10.36 min).

*The B-Type Cluster* (47 countries/territories): The strongest distinguishing feature of mobile gaming in B-Type Cluster countries is how uncommon it is: members of this group tend to have very low average amounts of mobile gaming per capita (mean = 3.6 h per capital per year). B-Type countries include India and mostly developing countries in Africa, Central and South America, Central and South Asia, and the Pacific Islands. As with other low-playtime per year clusters (e.g., F-Type), playtime tends to be relatively equally spread between gamers, with a moderately low proportion of playtime being drawn from the top 1%. India is a useful comparative example with the similarly populous nation of China from Cluster A-Type. Whilst active gamers in India tend to engage with games similarly in terms of play duration per day (13.4 min for India, 14.6 for China), the countries differ in terms of both hours of play per capita and inequality of play: Not only is play much less common in India than it is in China (7.1 vs 12.3 h of play per capita per year), but play inequality is considerably lower in India than in China: In China, 59.7% of playtime is concentrated within the top 1% of gamers—in India, just 37.4% is. However, for both of these countries, large unengaged segments of the population may bring down the duration of play per capita, opening up further research areas into the culture of play within these countries.

*The C-Type Cluster* (36 countries/territories) comprises a geographically diverse set of countries, including South American, Middle Eastern, Central Asian, and Caribbean nations. The most populous country in the cluster is Brazil. These territories exhibit comparable levels of playtime per capita to A-Type cluster members (mean = 26.3 h per capita per year). However, unlike A-Type countries, this moderate mobile gaming culture is not accompanied by such a strong concentration of playtime within a heavily engaged group.

*The D-Type Cluster* (27 countries/territories) is a grouping of developing countries across Africa, Asia, and Southern and Central America where mobile gaming is uncommon: these countries feature relatively small playtimes per day for active users (mean = 12.8 min), and low levels of playtime per capita (mean = 11.8 h per year). However, notedly, the populations of D-Type territories still spend markedly more time per year playing mobile games than representative members of the B-Type and F-Type clusters.

*The E-Type Cluster* (23 countries/territories) is characterised by very high duration per capita (mean = 44.05 h per year per capita) (exceeded only by the small but extreme H-Type Cluster) showing that mobile gaming tends to be more common in members of this cluster than almost anywhere else in the world. This omnipresence of mobile gaming is accompanied by a well-established layer of heavily engaged players, with a substantial proportion of overall playtime coming from the top 1% (mean = 51.58%). Average play per day in this cluster is variable, ranging 9.3 min per day for Greenland to 15.4 min per day for Thailand. Playtime per capita ranges from 35 h per year in Saint Lucia, 37 for the Russian Federation, 48 h for the USA to 50 h in Japan and up to 65 h in Guam. This cluster contains the USA, Canada, Japan, and the Russian Federation.

*The F-Type Cluster* (19 countries/territories), as with B-Type, is primarily characterised by a lack of overall mobile play: However, the scarcity of playtime of F-Type territories is even more extreme than in the B-Type cluster. Countries in this cluster have the lowest average levels of mobile playtime per capita anywhere in the world (mean = 2.03 h). The country with the least playtime per capita on the planet is Eritrea, at just over 20.4 s. What little play exists in F-Type territories tends to be evenly distributed between players: these countries also tend to have the lowest levels of play inequality of any territories on the planet (mean = 31.33%). F-Type members tend to be some of the world’s least developed countries, as well as countries whose infrastructure has been disrupted by conflict.

*The G-Type Cluster* (7 countries/territories) largely comprises Pacific Island states. Within G-Type territories, not many people play mobile games, but those that do tend to play more per day than in any other part of the world (mean = 30 min per day). Additionally, regardless of the duration per capita (for Papua New Guinea, only 0.84 h per capita per year to 38.3 h per year for Nauru), gameplay is relatively evenly distributed amongst its game-players: a relatively small proportion of playtime is concentrated within the top 1% (mean = 33.1%). The inclusion of Guinea (West Africa) and Vietnam (south-east Asia) is curious amongst a series of Pacific Island nations but is largely due to the even distribution of play across players within these territories (the top 1% accounting for only 26–43% compared with other clusters that average much higher) and the high average play per day.

*The H-Type Cluster* (4 countries/territories) is made up of wealthy east Asian territories with an on-average high standard of living: Singapore, South Korea, Macao SAR, and Hong Kong SAR. Gameplay in H-Type countries is extreme: it exhibits high inequality factors, a high average play per day (19.2 min), and the world’s highest duration per capita (mean = 70.7 h). This equates to the world's highest saturation of playtime per capita and is coupled with the presence of the world's most extreme gamers—the top 1% of players in these countries account for almost 58% of total playtime.

## Discussion

This paper provides a detailed view of the global distribution of mobile playtime, with data provided directly from the most widely used mobile games engine in the world. The world of mobile gaming is extremely diverse, but, as shown in the results of the cluster analysis, diverse in different ways from classic assumptions made about cultures of gaming. Informal theories of gaming, such as the dominant assumption that East Asian countries such as China^54,55^, Japan^56^, and South Korea^30,57^ are an exceptional group of gaming countries in terms of their playtime have been dispelled by this analysis. In fact, for mobile games at least, China is more similar to Europe in its gaming culture in terms of playtime, with only a mid-range play per day and low duration per capita despite a high concentration of play amongst the top 1% of gamers. Only South Korea conforms to these informal theories, with high levels on all three measures. Japan, on the other hand, is more similar to the US and Canada in terms of playtime. This understanding of similarities and differences in playtime is made possible by the multivariate approach taken here: whilst South Korea and Japan are both in the top five for hours of playtime per capita, this playtime is distributed differently across the two countries.

How do these results fit with prior work on cross-cultural differences in gaming? As noted throughout our introduction, prior cross-cultural work dealing with the variables examined limited datasets that tended towards self-report of engagement; convenience sampling; and limited geographical scopes. However, the results here partially replicate some of the patterns seen within the literature. For example, in^55^, researchers estimate that South Korea contains “the world’s most intense online gaming culture”, characterised by high prevalence rates of self-reported heavy engagement. This is mirrored by South Korea’s inclusion in our H-Type cluster, which is characterised by extreme levels of playtime inequality, play per capita and per user. Thus, this high playtime per capita may be reflective of gaming being highly accessible and normalised (e.g. ‘PC bang’ or Internet café culture in South Korea) (see e.g.^53,55,58^). Similarly, previous work has suggested a disproportionate prevalence of disordered gaming amongst adolescents in Hong Kong^59^. Whilst the research conducted here cannot speak to the problems associated with any observed playtime, we are able to observe that Hong Kong is characterised by high levels of play per user; and an inequal play culture in which significant proportions of playtime are concentrated within a heavy-playing subgroup. The results presented in^59^ do not contradict such a scenario. Prior results from the literature are not always in accordance with the data we see here. For example, in^10^, researchers highlight comparative differences in playtime between Japanese and British adolescent groups: In terms of self-report from a convenience sample, British adolescents tend to play less than adolescents from Japan. This is not mirrored in the results obtained here: The UK forms part of A-Type cluster, whilst Japan is grouped with countries like the USA and Canada in E-Type cluster. Whilst these clusters may differ substantially in terms of the commonness of gaming (i.e. playtime per capita), in terms of the duration of playtime per gamer they are similar. Such discords may be due to a number of factors—most notedly, the lack of representative samples in the literature and their reliance on self-report of play. However, it is important to note that this distinction may not simply be due to the methodology employed here—Coldwell and Kato studied a specific group (adolescents) at a specific time (2005). Thus, the expectation of concordance in this case may well be unfair. This presents a challenge for future work: In such a diverse and constantly-evolving field, replication of results may be challenging, undesirable, or impossible.

However, beyond simply replicating or not replicating patterns found in the literature, the results presented here also highlight more complex patterns in playtime developing throughout the world. To the best knowledge of the authors these have not been described previously, and should be important to explore in more detail in order to understand how (mobile) games are played globally and locally. To some extent, these clusters may reflect broad underpinning technological trends that relate to access to gaming: For example, self-report research has suggested that internet penetration rates and smartphone usage are low in many developing African nations (e.g. Senegal, Ghana, Nigeria); but high in economically developed European countries (e.g. UK, France, Spain)^60^. These underpinning trends in access may explain the cultures of gaming observed here: For example, the B-Type, D-Type, and F-Type clusters are characterised by low HPC (Hours of playtime per capita)—the fact that these clusters are dominated by developing countries may be associated with less developed national internet infrastructures. Similarly, the fact that economically developed European countries where smartphone usage is prevalent almost exclusively form the A-Type cluster may similarly be a product of underlying technological development levels. It is important to note that this aligns with prior theoretical perspectives within the gaming literature. For example, in^61^, researchers articulate the opinion that specific cultural and technological factors—such as strong broadband infrastructure combined with a collective culture—might lead to the uptake of specific forms of gaming technology (e.g. multiplayer gaming). Such factors could explain the results observed here.

However, broad infrastructural issues are not able to explain all the variation we observe between countries. One example of this is in the Caribbean islands that are included within the E-Type Cluster. Just like the USA, these island nations are typified by inequality in distribution of gaming and high play duration. These features might be explained by US tourism and military posts in the smaller islands, however, for the larger countries they may suggest lower levels of normalisation of mobile gameplay amongst the broader population, possibly as a consequence of socio-economic inequalities. Certainly, the Caribbean is increasing its capacity for mobile gameplay^62^, but the extremely large value for duration of play and the inequality of this spread seems to indicate more complex emergent patterns than simple increases in the volume of overall playtime. It is also intriguing to see the US, Russia, Canada, and Japan in a cluster together separate from other European and East Asian countries: more research into why these differences exist is needed, especially since South Korea, China, and Japan, as discussed, are assumed to have similar gaming cultures^63^. Likewise, while the US and Canada are seen to be culturally similar to each other given their proximity^64^, they are often also considered to be similar to European nations in terms of gaming populations and culture^28^. This analysis suggests that this may not be the case. Finally, there are more complex similarities between smaller nations that require further investigation: the similarity between Guinea, Vietnam, and a series of small Pacific Islands in the G-Type Cluster is typical of this. This similarity is not immediately understandable according to religio-cultural factors: most Pacific Islands are largely colonial Christianity-based and Indigenous Pacific Islander cultures, whereas Guinea is largely French-Muslim^65^. It is also not immediately understandable on the basis of economic factors: Vietnam and Guinea are largely agricultural nations, whereas Pacific Islands tend to rely on fishing and tourism^66,67^.

## Limitations

The work presented here relies on analysis of data from mobile games created with, or using components from, the Unity game engine, where Unity Analytics is enabled. While Unity is the dominant engine for mobile game development, and the dataset used here represents the first global analysis of player behaviour, conclusions cannot be drawn unilaterally from the data to the sum of all mobile games globally. While there is no obvious reason why games made using the Unity engine would not be representative of all mobile games made, there is no direct way to verify such an assumption, due to the lack of transparency in the games market. Furthermore, while mobile games comprise the largest segment of the global games market, it is possible that global playtime patterns for games running on PC or console platforms may differ from the results shown here. For example, while esports games have branched into mobile platforms, the genre continues to be dominated by PC games, and may show a different global and local pattern distribution than mobile games.

Relatedly, the selected features, while meaningful, comprise only one set of possible dimensions on which to cluster countries into different groups. This paper is by no means the limit of global differences in mobile play; rather, it is an attempt to highlight the depth of previously unexplored diversity. Future work will explore the aggregated data alongside other features associated with countries or regions, such as GDP and wealth.

An additional limitation of this work relates to the time period during which it was collected: October 2020–October 2021. During this period, towards the start of the COVID-19 pandemic, a diverse range of containment and closure policies (‘lockdowns’) were differentially affecting the activities that individuals could engage with across the globe^68^. There has been speculation in the literature that these changes may have differentially affected how playtime occurred during the period under test across the globe^69^. Indeed, recent research has shown that policy decisions such as school closures are able to important influence the volume of playtime occurring within a territory^70^. Thus, whilst the results obtained here may be able to characterise how playtime occurred across the globe during 2020–2021, their generalisability to earlier (and later) time periods is unclear. Substantial future work must explore the invariance of the clusters observed here across time.

The research conducted here is exploratory in nature: Rather than incisively address a specific research question, this study instead involves a more open-ended process of discovery. Thus, whilst we have broad research questions which we attempt to answer within this research, we have no formal hypotheses to test or theories to explicitly prove or disprove. As such, many of the results obtained here beg more questions than they provide answers: The discovery of distinct ways of engaging in gaming in various small island groups provide a good example of this. Whilst we believe that this is one of the key strengths of our approach, it also forms one of our key weaknesses: This work provides a foundation for future study, but does not explicitly falsify much pre-built theory. Further work must focus on exploring smaller, more specific theories about the nature of play—potentially in a localised manner. For example, such work could examine the relationships between wealth and play; or weather and play (e.g. does seasonality affect long hours of play). Such work may bear more direct and actionable theoretical and practical implications than the work conducted here.

The uniqueness of this data also creates difficulties for comparison. As stated in the introduction, to our knowledge there are no localized, national datasets or studies that describe mobile gaming culture with telemetry data. Instead, prior comparison relies to a large degree on smaller-scale studies, less formal stereotypes and community sentiment about features of a given country or region’s play. We have here proposed plausible explanations for the observed differences where possible and included reference to literature outside mobile games such as PC and console titles for completeness, but these should be interpreted only as hypotheses to be explored and (dis)confirmed in future work.

## Conclusions

The games industry is characterised by being global. Thanks to online distribution networks and services across mobile, PC and console platforms, games are competing internationally. However, there is currently a gap in the knowledge base regarding what this global marketplace looks like: how are games played? Where are there many players who are willing to put a lot of time into games? Information on these and other fundamental aspects of the games market have historically been difficult to come by.

Overall, this paper presents a world first analysis of gaming across the globe, and shows that rather than there being two homogenous groups in terms of playtime—East Asian gaming cultures and the rest of the world—there are a number of distinct and specific clusters. Many of these groupings have never been examined in the academic literature, responded to in a policy space, or been used to inform industry practice. By identifying these groups in a rigorous and transparent manner, this paper addresses the underlying issue that has prevented these kinds of analyses, allowing for better academic, industry, and policy research and decision-making. It is important to note that the insights presented here are only made possible by Unity Technologies opening their data stores to independent academic research. This kind of initiative is essential to the future of research into video games.

From the perspective of the mobile games industry, the results presented thus provide the beginning of a much more nuanced view of the global distribution of gameplay. Future work may integrate transactional data, genre information, temporal analysis and other contextual information, to provide industry stakeholders with better information regarding market analysis and benchmarking.

However, ultimately, behavioural data across platforms and games is not only useful for informing the design of game economies or market decisions, but also for creators generating new experiences for users, for researchers furthering the knowledge that we have about the video games industry, and for policymakers who wish to obtain a better understanding of how this industry fits in with local and global economies and societies. It is our hope that this work helps decision-making within these diverse communities.

## Data Availability

The supplementary materials contain the underlying data tables for each map and diagram in the article. Also included are the input feature tables for the cluster analysis presented, as well as the elbow plot/silhouette analysis. This allows for replication of the presented cluster analysis. Code for map plotting is also included as are high-resolution versions of the world maps included in this paper. The supplementary materials are available online here: https://osf.io/5rw47/?view_only=5b49379cefbd4b8ca31ab0fd5e868755.
